# Effect of Intralipid infusion on peripheral blood T cells and plasma cytokines in women undergoing assisted reproduction treatment

**DOI:** 10.1002/cti2.1328

**Published:** 2021-08-12

**Authors:** Kerrie L Foyle, David J Sharkey, Lachlan M Moldenhauer, Ella S Green, Jasmine J Wilson, Cassandra J Roccisano, M Louise Hull, Kelton P Tremellen, Sarah A Robertson

**Affiliations:** ^1^ Robinson Research Institute Adelaide Medical School University of Adelaide Adelaide SA Australia; ^2^ School of Pharmacy and Medical Sciences University of South Australia Adelaide SA Australia; ^3^ School of Medicine Flinders University Adelaide SA Australia; ^4^ Repromed Pty Ltd Dulwich SA Australia

**Keywords:** cytokines, embryo implantation, infertility, lipid emulsion, T cells, uterus

## Abstract

**Objectives:**

Intravenous infusion of Intralipid is an adjunct therapy in assisted reproduction treatment (ART) when immune‐associated infertility is suspected. Here, we evaluated the effect of Intralipid infusion on regulatory T cells (Treg cells), effector T cells and plasma cytokines in peripheral blood of women undertaking IVF.

**Methods:**

This prospective, observational pilot study assessed Intralipid infusion in 14 women exhibiting recurrent implantation failure, a clinical sign of immune‐associated infertility. Peripheral blood was collected immediately prior to and 7 days after intravenous administration of Intralipid. Plasma cytokines were measured by Luminex, and T‐cell subsets were analysed by flow cytometry.

**Results:**

A small increase in conventional CD8^+^ T cells occurred after Intralipid infusion, but no change was seen in CD4^+^ Treg cells, or naïve, memory or effector memory T cells. Proliferation marker Ki67, transcription factors Tbet and RORγt, and markers of suppressive capacity CTLA4 and HLA‐DR were unchanged. Dimensionality‐reduction analysis using the tSNE algorithm confirmed no phenotype shift within Treg cells or other T cells. Intralipid infusion increased plasma CCL2, CCL3, CXCL8, GM‐CSF, G‐CSF, IL‐6, IL‐21, TNF and VEGF.

**Conclusion:**

Intralipid infusion elicited elevated pro‐inflammatory cytokines, and a minor increase in CD8^+^ T cells, but no change in pro‐tolerogenic Treg cells. Notwithstanding the limitation of no placebo control, the results do not support Intralipid as a candidate intervention to attenuate the Treg cell response in women undergoing ART. Future placebo‐controlled studies are needed to confirm the potential efficacy and clinical significance of Intralipid in attenuating cytokine induction and circulating CD8^+^ T cells.

## Graphical abstract

The lipid emulsion Intralipid is commonly used as an adjunct therapy in assisted reproduction, but whether and how it improves immune adaptation for pregnancy is unknown. Here, we showed Intralipid infusion is associated with elevated plasma levels of several embryotrophic cytokines that may promote implantation, but no change in the abundance or phenotypes of regulatory T cells that mediate maternal immune tolerance.

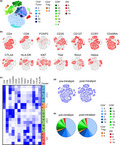

## Introduction

Recurrent implantation failure occurs when overtly healthy embryos fail to implant over the course of repeated IVF cycles. This condition affects at least 10% of infertile women seeking *in vitro* fertilisation (IVF) treatment,^1^ and is thought to reflect failure of the uterine endometrial lining to attain a sufficiently receptive state. Several factors likely contribute to impaired endometrial receptivity, but the uterine immune response and failure to acquire adequate immune tolerance are viewed as a likely cause in many women.^2^ For embryo implantation and formation of a robust placenta to support optimal fetal growth and development, a state of adaptive immune tolerance mediated by tolerogenic CD4^+^ regulatory T cells (Treg cells) is required.^3^ Tregs are well known for their capacity to limit excessive inflammation and recalibrate tissue homeostasis after insult or injury, as well as to suppress effector T‐cell reactions to self or non‐self antigens.^4^, ^5^ In fetal tolerance, CD4^+^ Treg cells are required to suppress inflammation, prevent effector T‐cell generation and support uterine vascular adaptations that underpin placental development.^4^, ^5^, ^6^

During early pregnancy, T cells comprise 10–20% of uterine immune cells.^7^ Many of these are CD8^+^ T cells, including regulatory subsets.^8^, ^9^ Amongst the CD4^+^ T cells, about 10–30% express the Treg transcription factor FOXP3, a substantial enrichment compared with peripheral blood.^10^, ^11^, ^12^ The majority of T cells in the human decidua have a memory phenotype (CD45RA^−^ or CD45RO^+^).^9^, ^13^ Decidual Th1 cell frequencies are moderately elevated, while Th17 and Th2 cells are generally not enriched, reflecting a mild inflammatory environment modulated by Treg cells.^11^, ^14^ Indeed, sufficient Treg cells must be present in the endometrium to suppress inflammation and allow successful embryo implantation to occur.^15^

As well as T cells, there are abundant populations of innate immune cells in the decidua, particularly macrophages,^16^ dendritic cells^17^ and a unique population of NK cells with a CD56^hi^CD57^lo^ phenotype (uterine NK cells or uNK cells).^18^ Treg cells may be important regulators of uNK phenotype and function at implantation,^19^ since Treg cells control IL‐15 release from DCs^20^ and suppress uNK cytolytic activity.^21^ The abundance, phenotype and function of these immune cell networks are modulated by a complex array of pro‐inflammatory and immune‐modulatory cytokines and chemokines in the uterine endometrium.^22^, ^23^, ^24^ Shifts in the endometrial cytokine balance can impact the maternal immune response and modify the events of placental development, to either promote or compromise progression of a viable pregnancy.^3^

Treg cell‐mediated tolerance arises in the pre‐implantation phase of early pregnancy and depends on interactions between maternal, paternal and conceptus‐derived signals at the mucosal surface of the uterine endometrium.^3^ Uterine Treg cells are recruited from precursors circulating in the peripheral blood, after proliferation and expansion in lymph nodes draining the uterus.^3^ Studies in cycling women show that peripheral blood and uterine Tregs fluctuate over the course of the menstrual cycle in response to regulation by oestrogen and progesterone, increasing in the peri‐ovulatory and early luteal phase in readiness for embryo implantation.^3^, ^25^ Several studies report that recurrent implantation failure is associated with insufficient Treg cells in the uterine mucosa or decidual lining.^26^, ^27^, ^28^ This is reflected in fewer numbers of these cells in the peripheral blood,^27^ and IVF success correlates with circulating levels of CD4^+^CD25^+^FOXP3^+^ cells,^29^, ^30^ suggesting a systemic defect. Associations between reduced fertility and fewer Tregs in asthma, allergy and autoimmune disease support systemic Treg cell dysfunction being an underlying cause.^31^ This has raised the imperative to develop and evaluate interventions that boost Treg cells in women experiencing recurrent implantation failure with an immune aetiology.^2^

Intralipid is a sterile lipid emulsion of polyunsaturated fatty acids derived from soya bean oil and egg yolk phospholipids used for parenteral nutritional support. Lipid emulsion formulations are demonstrated to have a variety of immune‐modulatory, anti‐inflammatory and antioxidative properties, depending on the specific composition and particularly the balance of omega‐3 and omega‐6 polyunsaturated fatty acid (PUFA) content.^32^ Many reproductive medicine clinics offer Intralipid as a so‐called ‘add‐on’ therapy, often at considerable cost to patients.^33^, ^34^ However, routine use of Intralipid in IVF is not supported by robust evidence of safety and efficacy.^35^ Several small clinical studies suggest that Intralipid therapy may exert therapeutic benefit for women experiencing repeated implantation failure,^36^, ^37^, ^38^, ^39^, ^40^ but this is not a consistent finding.^35^, ^41^, ^42^ A recent meta‐analysis concluded a modest positive benefit for women with implantation failure, but because of the small sample size, it is cautioned that more evidence is needed before routine application can be recommended.^43^ Although this potential benefit is presumed to occur through effects on immune adaptation to pregnancy, the biological rationale and mechanism of action in reproductive‐aged women is not clear,^43^ and whether Intralipid might affect Treg cell‐mediated tolerance is unknown.

While it has been claimed that Intralipid may attenuate aberrant populations of uNK cells, this is based on a single report that Intralipid reduces NK cell cytotoxic activity *in vitro*,^44^ and only one study has reported that Intralipid may decrease NK cell frequency in peripheral blood.^39^ Furthermore, although increased NK cell activity is regularly linked with pregnancy failure, the significance of this is disputed.^45^ Based on studies of the effects of lipid emulsions in other clinical settings, there is a biological rationale to suggest that Intralipid might alter the abundance or phenotype of T cells important for uterine receptivity.^32^, ^46^, ^47^ However, no studies to date have examined the effects of Intralipid on uterine or peripheral blood T cells in reproductive‐aged women, despite their importance in maternal immune tolerance of pregnancy.

To explore the possibility that T cells might be responsive to Intralipid infusion in women with recurrent implantation failure, we examined T cells in peripheral blood taken from women before and after Intralipid infusion in women undertaking IVF. Specifically, we utilised multi‐parameter analysis to allow detailed characterisation of the effects of intravenous Intralipid on beneficial anti‐inflammatory Treg cells, and potentially detrimental T effector immunity, or to compare T‐cell populations and cytokine and chemokine abundance before and after Intralipid treatment.

## Results

### Participant characteristics, clinical outcome, CRP and white blood cell parameters

The study participants included 14 women, with a mean ± SD (range) age of 35.8 ± 4.5 (27–43) years, BMI of 25.7 ± 7.4 (18.4–41.4), and total number of embryos transferred in prior ART cycles of 4.6 ± 2.0 (2–8) (Table 1). IVF treatment outcomes in the study cycle showed embryo implantation occurred in 8 women as indicated by a positive hCG test, leading to live birth in 5 of 14, early miscarriage (termed ‘early biochemical miscarriage’, EBM) in 2 women and 1 ectopic pregnancy. The remaining 6 women experienced implantation failure (Table 1). Intralipid did not alter the relative abundance of total white blood cells, neutrophils, lymphocytes, monocytes, eosinophils or basophils in peripheral blood, as indicated by comparison of the pre‐Intralipid and post‐Intralipid samples (Supplementary table 2). C‐reactive protein (CRP), a biomarker of systemic inflammation, was also not changed after Intralipid infusion (median (range) = 2.3 (0–20) before and 5.1 (0–47) after Intralipid).

**Table 1:** Study participant characteristics

Participant	Age	BMI	Total embryos transferred^a^	Embryo implantation	Pregnancy outcome
A	40	36.2	6	Yes	EBM^a^
B	37	25.1	3	Yes	Live birth
C	37	19.6	5	No	No
D	27	18.4	2	Yes	Ectopic
E	38	N/A	6	No	No
F	43	32.1	4	No	No
G	37	24.6	7	Yes	Live birth
H	28	19.0	3	Yes	Live birth
I	34	29.9	3	Yes	Live birth
J	35	18.5	2	No	No
K	33	41.4	4	Yes	EBM
L	35	19.6	4	No	No
M	34	26.5	7	Yes	Live birth
N	43	22.9	8	No	No
all	35.8 ± 4.7	25.7 ± 7.4	4.6 ± 2.0		LBR = 35.7%

BMI, body mass index; EBM, early biochemical miscarriage; Ectopic, ectopic pregnancy; LBR, live birth rate.

Summary data are mean ± standard deviation.

a Total embryos transferred = sum of prior ART cycles.

### Increased CD8^+^ Tconv cells but not CD4^+^ T cells or Tregs after Intralipid by 2D flow cytometry

**Figure 1:**
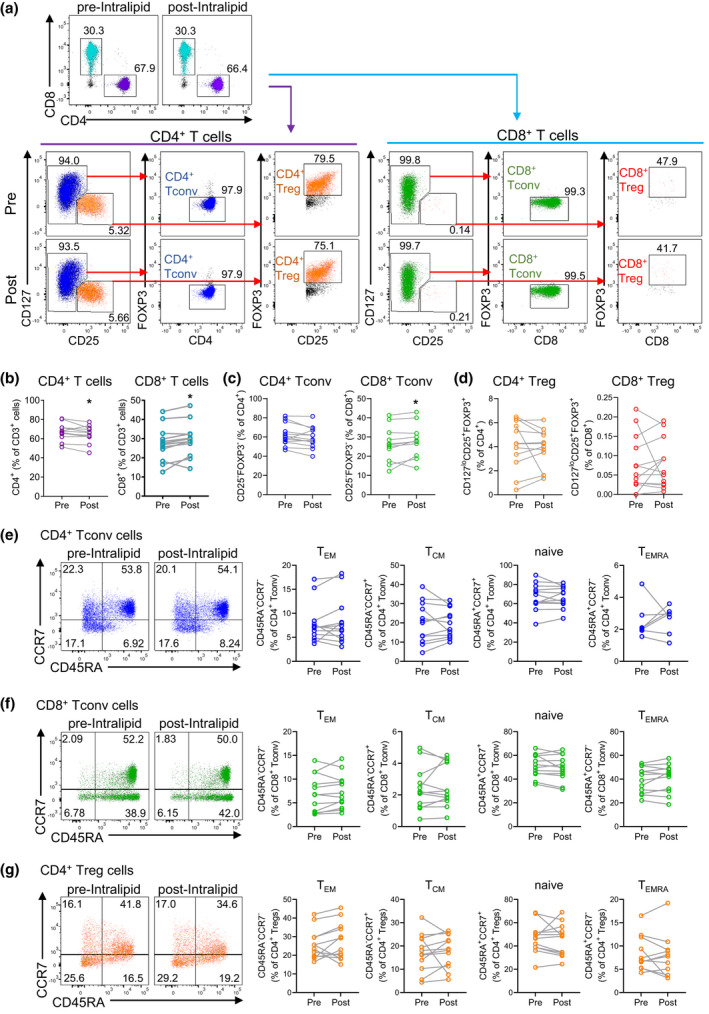
T‐cell populations in peripheral blood before and after Intralipid treatment. Peripheral blood samples obtained before and after Intralipid treatment were analysed by flow cytometry (N = 28 paired samples from N = 14 women). Differences in T‐cell parameters between pre‐ and post‐Intralipid samples were evaluated by the Wilcoxon matched‐pairs signed rank test. **(a)** Representative flow cytometry showing the gating of total CD4^+^ and CD8^+^ T cells (top row) and their conventional (Tconv) and regulatory (Treg) subpopulations (second and third rows) pre‐ and post‐Intralipid treatment. **(b–d)** Proportion of total CD4^+^ and CD8^+^ T cells **(b)**, CD4^+^ Tconv and CD8^+^ Tconv **(c)**, and CD4^+^ Treg and CD8^+^ Tregs **(d)** in peripheral blood pre‐ and post‐Intralipid treatment. **(e–g)** Effector and memory T‐cell subsets in CD4^+^ Tconv cells (blue; **e**), CD8^+^ Tconv cells (green; **f**) and CD4^+^ Tregs (orange; **g**). T_CM_ = central memory T cells; T_EM_ = effector memory T cells; T_EMRA_ = T effector memory cells expressing CD45RA. *P < 0.05.

To investigate the effect of Intralipid treatment on peripheral blood T cells, cryopreserved peripheral blood mononuclear cells (PBMCs) from pre‐ and post‐Intralipid blood samples were thawed, stained with antibodies against a panel of T‐cell markers and analysed by flow cytometry. Initially, flow cytometric data analysis utilised a conventional manual gating (2D) approach. A small reduction in the proportion of CD4^+^ T cells amongst total CD3^+^ T cells was present after Intralipid treatment, from a median of 67.2% of CD3^+^ T cells at baseline to 65.0% after Intralipid (*P* = 0.027), although neither conventional CD4^+^FOXP3^−^ T cells (Tconv) nor CD4^+^CD25^+^CD127^lo^FOXP3^+^ Treg cells were significantly different (Figure 1a–d). Conversely, total CD8^+^ T cells amongst CD3^+^ T cells were proportionally increased after Intralipid, because of a small increase in CD8^+^FOXP3^−^ Tconv cells from a median of 27.2% to 29.3% post‐Intralipid (*P* = 0.026). CD8^+^CD25^+^CD127^lo^FOXP3^+^ Treg cells comprised only a small proportion of T cells (0.06%) and were unchanged by Intralipid treatment. Notably, analysis of CD4^+^ FOXP3^+^ and CD8^+^FOXP3^+^ T cells independently of CD25 and CD127 expression yielded a similar result of no effect of Intralipid treatment (data not shown).

### The phenotypes of T cells are unaffected by Intralipid treatment

CD4^+^ Tconv cells were mostly naïve (65.6%) and central memory T (T_CM_) cells (20.6%), and there was no change in their frequency or in effector memory T cells (T_EM_) or T effector memory re‐expressing CD45RA (T_EMRA_) cells post‐Intralipid treatment (Figure 1e). Likewise, the proportions of effector and memory subsets of CD8^+^ Tconv cells were also unchanged by Intralipid treatment and were mostly of a naïve (50.9%) or T_EMRA_ phenotype (41.4%) (Figure 1f). The effector and memory subsets of Tregs were also analysed and found not to be changed by Intralipid treatment (Figure 1g). Most Tregs exhibited a naïve phenotype (46.7%) although some T_CM_ (17.3%), T_EM_ (24.5%) and T_EMRA_ (7.2%) were also present.

**Figure 2:**
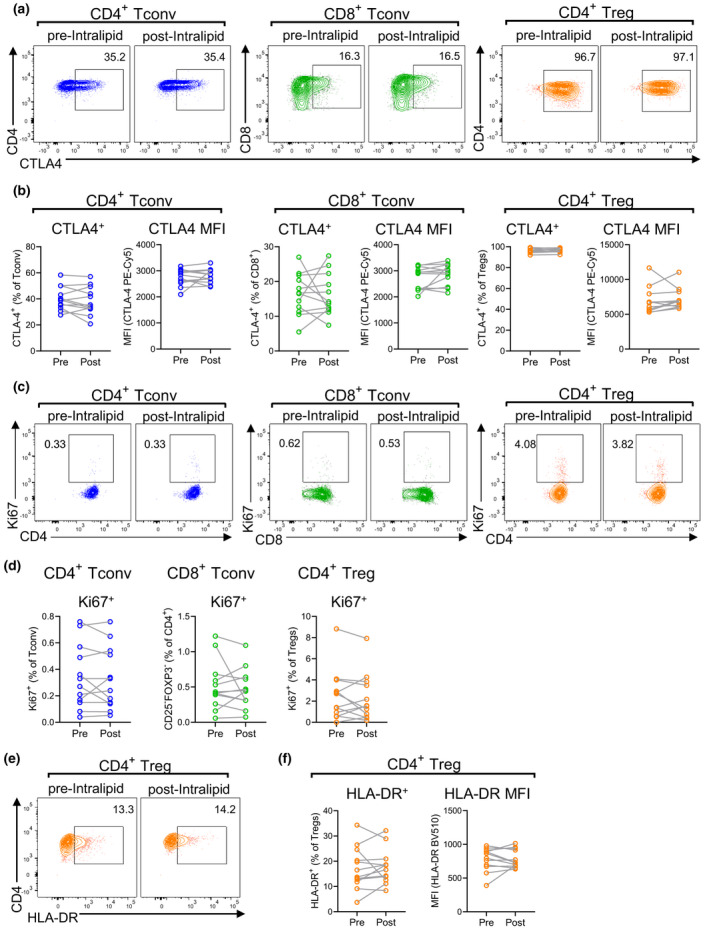
Expression of CTLA4, Ki67 and HLA‐DR by T‐cell populations before and after Intralipid treatment. Peripheral blood samples obtained before and after Intralipid treatment were analysed by flow cytometry (N = 28 paired samples from N = 14 women). Differences in T‐cell parameters between pre‐ and post‐Intralipid samples were evaluated by the Wilcoxon matched‐pairs signed rank test. **(a)** Representative flow cytometry and graphs **(b)** of proportion of CTLA4‐expressing conventional CD4^+^ T cells, conventional CD8^+^ T cells and CD4^+^ Tregs. **(c)** Representative flow cytometry and proportions **(d)** of proliferating T cells (Ki67^+^). **(e)** Representative flow cytometry and proportions **(f)** of HLA‐DR expression by CD4^+^ Tregs.

There was no difference in the frequency of CD4^+^ Tconv, CD8^+^ Tconv or Tregs that expressed Tbet or RORγt, indicating no bias towards Th1 or Th17 immunity (Supplementary figure 1a). No change was found in the expression of the exhaustion marker CTLA4 by CD4^+^ and CD8^+^ Tconv cells, as measured by both percentage of CTLA4^+^ cells and mean fluorescence index (MFI) (Figure 2a, b). Treg expression of CTLA4, which is associated with immunosuppressive function, was also similar in pre‐ and post‐Intralipid samples. HLA‐DR is another molecule expressed by some Tregs, and its increased expression level is associated with suppressive phenotype.^48^ Like CTLA4, no difference in Treg expression of HLA‐DR was detected after Intralipid treatment (Figure 2e, f).

Few Tconv cells were proliferating as measured by Ki67 expression, representing only 0.30% and 0.42% of CD4^+^ and CD8^+^ Tconv, respectively (Figure 2c, d). Comparatively, a higher proportion of CD4^+^ Tregs (2.05%) were proliferating (Figure 2c, d), but no differences in proliferation of any of these T‐cell subsets were detected after Intralipid treatment.

### tSNE clustering analysis showed no change in T‐cell subsets after Intralipid

**Figure 3:**
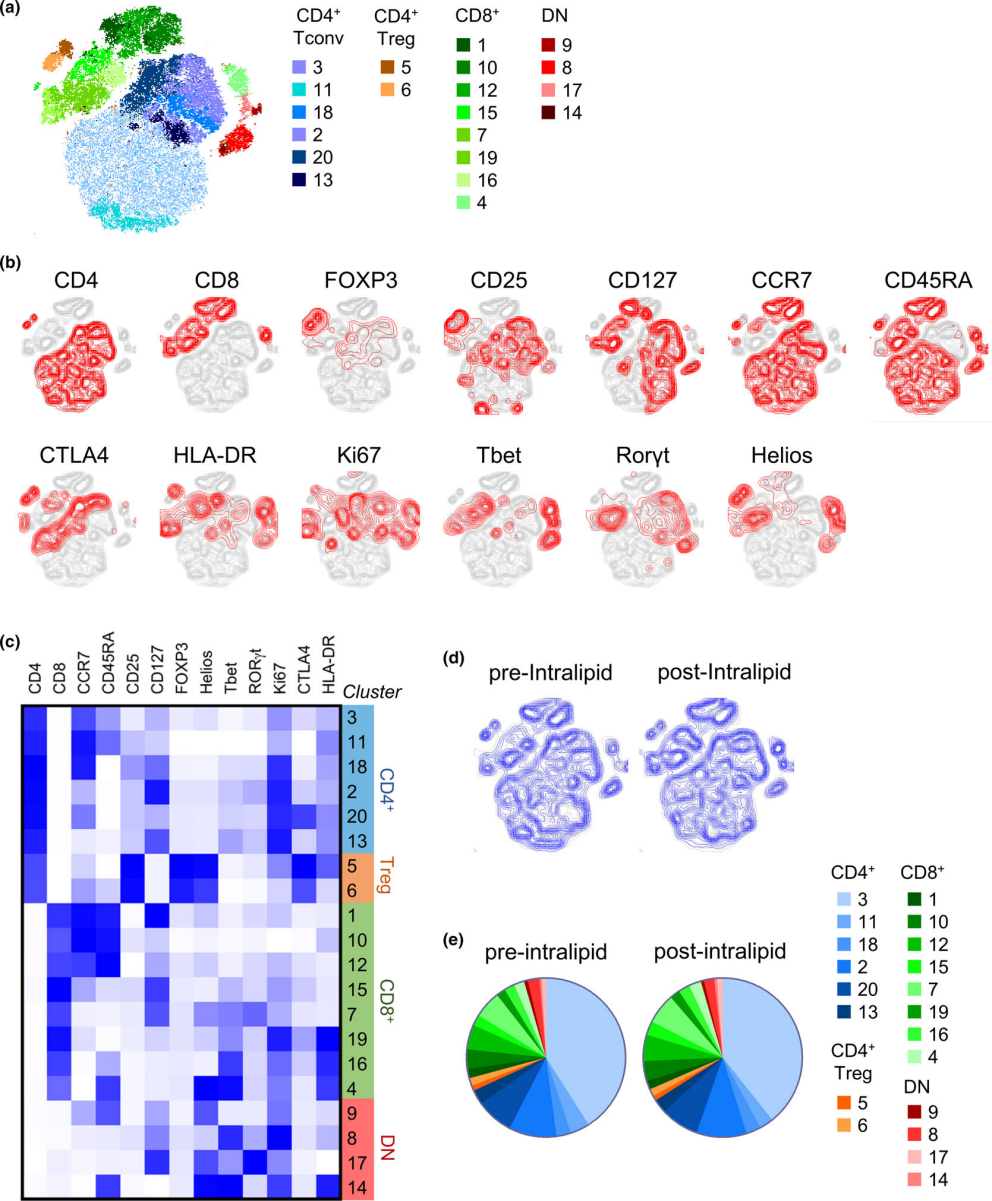
Effect of Intralipid treatment on CD3^+^ T cells in peripheral blood as assessed by t‐distributed stochastic neighbour embedding (tSNE). Peripheral blood samples obtained before and after Intralipid treatment were analysed by flow cytometry (N = 28 paired samples from N = 14 women). A downsample of 3500 CD3^+^ T cells per sample was concatenated and clustered using Xshift and tSNE algorithms. **(a)** 2D tSNE representation of cell clustering. Each colour represents a different T‐cell cluster defined by the Xshift algorithm. **(b)** Expression of each marker mapped to the tSNE plot. Cells were manually gated for positive expression of each marker, then mapped to the tSNE plot. **(c)** Heatmap of expression of each marker by the Xshift‐defined cell clusters. The clusters have been grouped into families of CD4^+^, CD8^+^ and CD4^‐^CD8^‐^ clusters based on the presence or absence of CD4 or CD8 in the first 2 columns of the heatmap. Blue indicates high expression level of the molecule relative to other clusters, and white indicates no expression. **(d)** tSNE plots of CD3^+^ cells from IVF patients pre‐ and post‐Intralipid treatment. **(e)** Proportion of each cell cluster within CD3^+^ T cells in IVF patients pre‐ and post‐Intralipid treatment.

Additionally, flow cytometric data were analysed using an unbiased, dimensionality‐reducing and clustering algorithm to investigate differences between T cells in pre‐ versus post‐Intralipid blood samples. A sample of 3500 live CD3^+^ T cells was randomly selected from each pre‐ and post‐Intralipid PBMC sample and the clustering algorithm Xshift employed to define T‐cell subpopulations in an unbiased manner. The tSNE algorithm was applied to depict the 20 cell clusters that were identified (Figure 3a). The expression of each of the markers by the CD3^+^ T cells was assessed, and CD4^+^ and CD8^+^ T cells were clustered into two main areas each (Figure 3b). A heatmap was generated detailing the expression level of each cell marker by the different T‐cell clusters (Figure 3c). From this heatmap, CD4^+^ and CD8^+^ T cells and Tregs were manually annotated. PBMCs isolated from women following Intralipid treatment showed a similar T‐cell composition to baseline PBMCs (Figure 3d, e), and there was no difference in the Xshift‐defined T‐cell populations between the pre‐ and post‐Intralipid samples as determined by the Wilcoxon matched‐pairs signed rank tests (Supplementary table 3). Thus, unbiased cell clustering analysis of the flow cytometric data revealed no changes to T cells in the blood of women undergoing IVF following Intralipid treatment.

### Increased cytokines and chemokines in post‐Intralipid plasma

**Figure 4:**
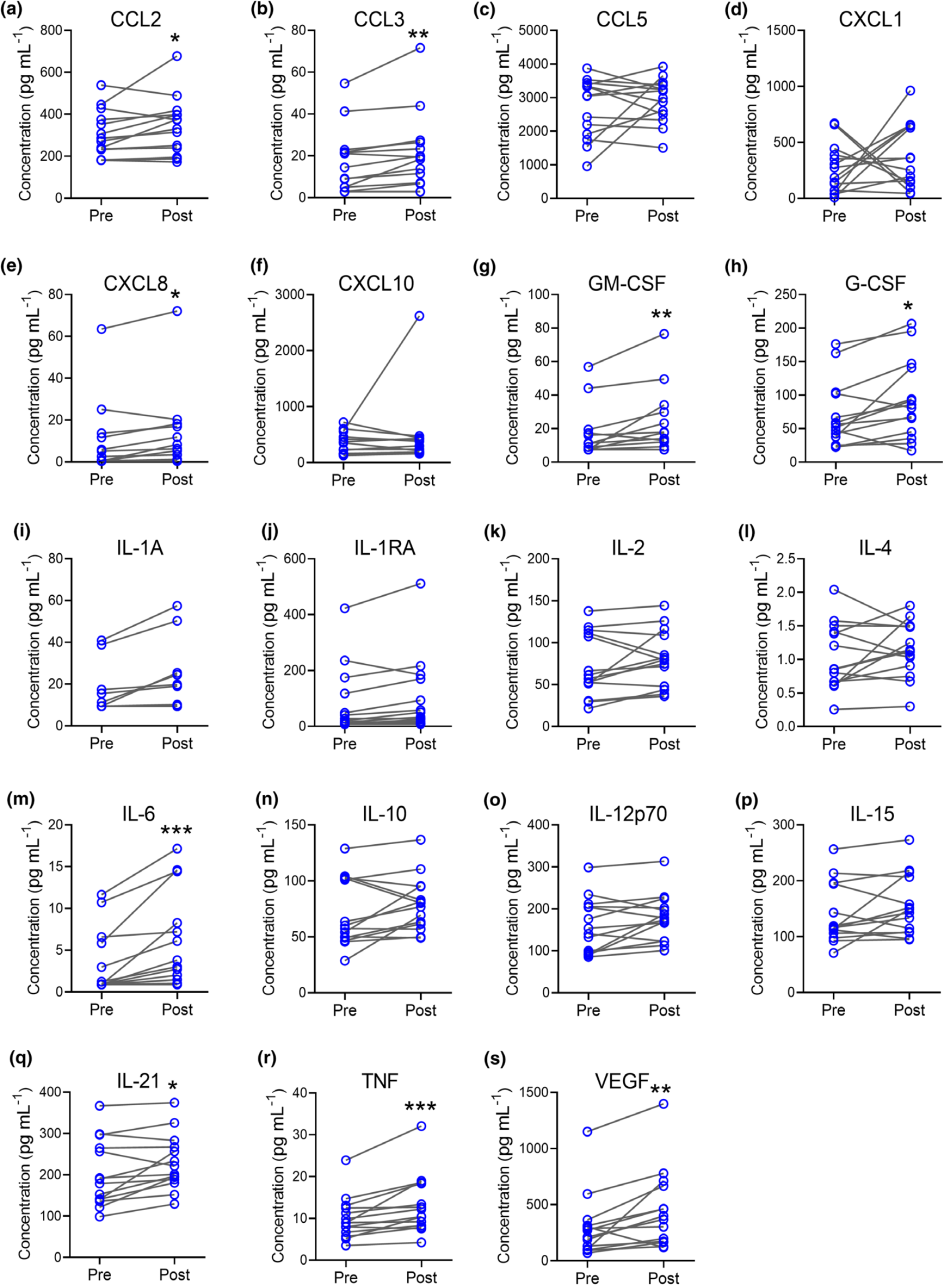
Effect of Intralipid treatment on plasma cytokine and chemokine levels. Plasma prepared from peripheral blood obtained before and after Intralipid treatment was analysed by Milliplex MAP Luminex microbead assay (N = 28 paired samples from N = 14 women) to quantify CCL2 **(a)**, CCL3 **(b)**, CCL5 **(c)**, CXCL1 **(d)**, CXCL8 **(e)**, CXCL10 **(f)**, GM‐CSF **(g)**, G‐CSF **(h)**, IL‐1A **(i)**, IL‐1RA **(j),** IL‐2 **(k)**, IL‐4 **(l)**, IL‐6 **(m)**, IL‐10 **(n)**, IL‐12p70 **(o)**, IL‐15 **(p),** IL‐21 **(q),** TNF **(r)** and VEGF **(s)**. Differences in cytokine and chemokine concentrations between pre‐ and post‐Intralipid samples were evaluated by the Wilcoxon matched‐pairs signed rank test. *P < 0.05, **P < 0.01 and ***P < 0.005.

Cytokines and chemokines in peripheral blood plasma are an additional measure of systemic immune status. Luminex microbead assay was used to measure the concentrations in plasma of an array of pro‐ and anti‐inflammatory cytokines and chemokines with established roles in the innate and adaptive immune response. Intralipid infusion was associated with increased plasma CCL2, CCL3, GM‐CSF, G‐CSF, IL‐6, IL‐21 and TNF (Figure 4a, b, e, g, h, m, q, r and Supplementary table 4). Vascular endothelial growth factor (VEGF), which has anti‐inflammatory and pro‐angiogenic properties, was also increased (Figure 4s). Other cytokines and chemokines including IL‐1‐receptor alpha (IL‐1Ra), IL‐2, IL‐4, IL‐10, IL‐12p70, IL‐15, CCL5, CXCL1 and CXCL10 were unchanged (Figure 4). When effects of Intralipid were analysed according to implantation success, significant increases in CCL3, GM‐CSF, IL‐6, IL‐10, IL‐15, IL‐21 and VEGF were seen in the post‐Intralipid versus pre‐Intralipid plasma samples in the subset of women where embryo implantation occurred, but not in women where embryo implantation did not occur (Table 2, Supplementary figure 2). However, there was no association between implantation success or later live birth and the levels of plasma cytokines prior to or after Intralipid treatment, or the degree of cytokine increase between the pre‐Intralipid and post‐Intralipid time points (Table 2, Supplementary figure 2).

**Table 2:** Plasma cytokine and chemokine concentrations before and after Intralipid administration, according to clinical outcome of ‘embryo implantation’ versus ‘no embryo implantation’

Cytokine/chemokine	No embryo implantation (*n* = 6)		Embryo implantation (*n* = 8)^a^	
	pre‐Intralipid (pg mL^−1^) Median (range)	post‐Intralipid (pg mL^−1^) Median (range)	pre‐Intralipid (pg mL^−1^) Median (range)	post‐Intralipid (pg mL^−1^) Median (range)^b^
CCL2	256.0 (181.4–352.1)	353.1 (186.9–418.5)*	330.2 (181.5–538.9)	344.3 (173.2–677.4)
CCL3	15.5 (2.9–54.7)	19.0 (2.9–71.6)	11.7 (2.9–41.3)	18.9 (6.7–43.9)*
CCL5	2738.3 (1745.9–3528.3)	2770.0 (1506.4–3442.9)	2624.9 (958.8–3874.4)	3113.8 (2507.9–3922.5)
CXCL1	245.7 (40.7–444.2)	505.4 (43.8–963.7)	136.6 (9.9–670.6)	308.2 (50.1–649.2)
CXCL8	1.7 (0.4–13.7)	1.9 (0.4–18.2)	3.2 (0.4–63.5)	10.1 (0.9–72.11)
CXCL10	194.8 (142.5–458.1)	215.0 (182.9–380.2)	455.7 (129.5–721.0)	325.6 (158.4–2620.2)
G‐CSF	45.0 (25.1–162.6)	66.8 (17.3–206.5)	80.5 (22.6–176.2)	92.9 (35.4–195.2)
GM‐CSF	9.4 (7.5–56.9)	13.5 (7.5–76.5)	13.6 (7.5–44.1)	20.5 (7.5–49.6)*
IL‐1A	9.4 (9.4–40.9)	9.4 (9.4–57.3)	10.3 (9.4–38.9)	15.0 (9.4–50.3)
IL‐1RA	21.6 (8.3–174.7)	20.4 (8.3–216.5)	33.9 (8.3–236.0)	71.6 (17.6–511.4)
IL‐2	64.0 (29.8–111.2)	79.5 (36.2–85.3)	83.7 (21.8–137.7)	112.7 (38.6–144.3)*
IL‐4	1.1 (0.7–2.0)	1.1 (0.7–1.8)	1.0 (0.3–1.6)	1.4 (0.3–1.6)
IL‐6	0.9 (0.9–11.6)	1.8 (0.9–17.2)	2.1 (0.9–10.7)	5.0 (2.7–14.4)**
IL‐10	60.6 (46.7–104.2)	69.2 (49.1–83.0)	80.5 (28.7–128.9)	95.3 (49.8–136.8)*
IL‐12p70	164.9 (132.8–234.1)	176.2 (112.7–224.4)	172.9 (85.5–298.9)	194.9 (100.9–313.5)
IL‐15	117.0 (92.9–194.7)	129.0 (95.1–157.9)	155.0 (70.8–256.7)	211.3 (96.0–273.5)*
IL‐21	190.9 (136.3–298.4)	211.3 (152.0–282.9)	221.8 (98.8–366.8)	261.8 (129.6–374.4)**
TNF	7.8 (3.5–23.9)	8.7 (4.2–32.1)*	10.6 (5.2–14.7)	12.8 (7.6–18.6)*
VEGF	196.1 (94.7–1150.4)	250.9 (119.2–1398.4)	292.7 (69.4–595.2)	462.4 (172.2–779.3)**

Cytokine levels prior to and after Intralipid treatment were not different between the ‘embryo implantation’, and ‘no embryo implantation’ groups (Sidak’s *t*‐test). Refer to text for additional details. See Supplemental figure 2 for individual values, and Supplemental table 4 for values when embryo implantation and no embryo implantation groups are combined.

a Differences between pre‐ and post‐Intralipid samples were analysed by the Wilcoxon matched‐pairs signed rank test. *P < 0.05 and **P < 0.01.

b The ‘embryo implantation’ group includes live birth (*n* = 5), biochemical pregnancy (*n* = 2) and ectopic pregnancy (*n* = 1).

## Discussion

This study provides the first detailed analysis of the effects of Intralipid on circulating T cells in women undergoing assisted reproduction treatment. In broad terms, we found no increase in Treg cells, no substantial shift in the balance of CD4^+^ or CD8^+^ regulatory to conventional T cells, and no indication of altered phenotype in Treg cells or other T‐cell subsets, which were attributable to Intralipid treatment. In contrast, Intralipid infusion was associated with changes in peripheral blood plasma cytokines, notably elevated CCL2, CCL3, GM‐CSF, G‐CSF, IL‐6, IL‐21 and TNF, compared with baseline concentrations. This could reflect an effect of Intralipid on circulating immune cells, but, given the pattern of cytokines seen, is unlikely to be because of T‐cell activation, and is more likely associated with innate immune cells or non‐leucocyte sources. The data therefore do not support an impact of Intralipid on Treg cells or other elements of the adaptive immune response that would be sufficient to influence embryo implantation or the likelihood of successful pregnancy.

An important consideration in the interpretation of the data is the lack of a no‐Intralipid or saline infusion control to compare the results from Intralipid‐treated women. Thus, we are unable to conclusively attribute the minor changes in CD8^+^ T cells or shift in plasma cytokines to Intralipid, since other factors might also contribute. As well as endogenous cycle‐related changes in ovarian steroid hormones, all women were administered exogenous progesterone according to standard clinical IVF protocols.^49^ No other drugs were administered. While elevated progesterone of endogenous or exogenous origin can alter peripheral blood cytokine and immune parameters, many published studies show that the impact is to reduce pro‐inflammatory cytokines and effector T cells, in line with the anti‐inflammatory effect of this hormone.^50^, ^51^ These considerations support the prospect that the observed immune changes are because of Intralipid, but further studies are required to prove this.

While CD4^+^ T cells were unchanged, a small 8% increase in median relative proportion of CD8^+^ T cells was seen after Intralipid treatment, due mainly to increased CD8^+^ Tconv as opposed to CD8^+^ Treg cells. Whether this elevation in Tconv cells is beyond normal fluctuations seen over the course of the menstrual cycle is not certain, but notably, effector T cells are generally diminished, not elevated, in the luteal phase.^50^ Since no differences were found within the CD8^+^ Tconv cell phenotype, the physiological significance in relation to availability of CD8^+^ T cells for recruitment into the uterus is not known. Compared with circulating CD8^+^ T cells, decidual CD8^+^ T cells display a more differentiated, T_EM_ phenotype and can recognise fetal antigens, although they express lower cytolytic molecules perforin and granzymes.^9^ Future studies to evaluate the impact of Intralipid on uterine immune cells would be required to determine the relevance of the peripheral blood changes to potential changes in the uterus. Even if the small increase in CD8^+^ Tconv cells was mirrored in the decidua, any threat posed to reproductive success would likely be attenuated by uterine Treg cell populations and high expression of the co‐inhibitory molecule PD‐1, which together keep effector functions of decidual CD8^+^ Tconv cells in check.^52^

Although the current study was not powered to investigate relationships between IVF treatment outcome and immune parameters, it was interesting to consider whether the minor T‐cell changes were evident to a greater or lesser degree in women who progressed to pregnancy and/or live birth. In an exploratory analysis, we compared T‐cell data from the 6 women who experienced implantation failure to data from the 8 women where embryo implantation occurred, and data from the 5 women where live birth was achieved. This analysis showed no significant difference in the post‐Intralipid CD8^+^ T cells, or other T‐cell proportions or phenotypes, and no indication of a greater change compared with baseline in these subgroups.

The lack of effect of Intralipid infusion on Treg cells was in contrast to previous studies showing that lipid emulsions can alter T‐cell parameters *in vitro* and *in vivo*. Different effects of lipid emulsions are demonstrated, depending on the lipid composition and notably the balance of omega‐3 and omega‐6 PUFA.^32^, ^53^ As a soya bean‐based emulsion, Intralipid has a 7:1 omega‐6: omega‐3 PUFA ratio,^54^ is generally thought to be immunosuppressive and is able to inhibit lymphocyte, macrophage and neutrophil functions.^32^, ^53^ *In vitro* experiments have shown that T lymphocyte proliferation is suppressed in the presence of low doses of Intralipid and related emulsions,^46^ consistent with a potential effect on Treg cells. Treg cells are known to have specific lipid requirements, and FOXP3 induction can be suppressed when triglyceride uptake and metabolism is impaired.^47^ These immune‐regulatory properties are mediated through impacts on eicosanoid synthesis and direct effects on cytokine synthesis,^55^ potentially via regulation of the peroxisome proliferator‐activated receptors (PPARs), which modulate the expression of genes involved in the immune and inflammatory response, for example by antagonising NFκB signalling.^56^

Whether or how the induction of CCL2, CCL3, GM‐CSF, G‐CSF, IL‐6, IL‐21 and TNF after Intralipid infusion might have a bearing on embryo implantation is not clear. Several previous studies indicate a local inflammation‐like response in the uterine endometrium is required at embryo implantation, in order to facilitate maternal immune adaptation for pregnancy.^3^, ^22^, ^23^ The cytokines that were elevated after Intralipid infusion each has pro‐inflammatory and immune‐regulatory properties, and all are expressed in the endometrium during early pregnancy.^22^, ^24^, ^57^ This local inflammatory state is accompanied by systemic indicators of inflammatory activation, as evidenced by higher plasma CRP levels in pregnant than in non‐pregnant IVF patients detectable from 10 days after embryo transfer.^58^ Elevated CRP associated with implantation success was not detected in the current study, presumably because of the earlier sampling time, and the small number of women evaluated.

In the absence of a no‐Intralipid control group, it is not possible to attribute conclusively the elevated plasma cytokines to Intralipid treatment. However, for all cytokines other than TNF, the cytokine induction after Intralipid was substantially greater than the expected increase because of cycle‐related fluctuations.^59^, ^60^, ^61^, ^62^ Intralipid is known to exert pro‐inflammatory effects by virtue of its high content of omega‐6 PUFAs, which metabolise to 2‐series prostaglandins and thromboxanes, and 4‐series leukotrienes.^54^ In line with the current data, a previous study reported that Intralipid infusion acts to elevate TNF and IL‐6 in response to low‐dose endotoxaemia in humans.^63^ It is possible that local synthesis in the uterus because of embryo implantation contributed to elevated plasma cytokines after Intralipid, especially given that increases were more consistently seen in women with successful implantation. However, this is unlikely given that the post‐Intralipid sample was recovered so early after implantation commences, and before extensive trophoblast invasion could occur. It is possible that differential degrees of cytokine induction reflect varying levels of systemic responsiveness to Intralipid stimulation. Therefore, possible implications for fertility of the elevated changes in cytokine and chemokine levels in post‐Intralipid plasma samples are discussed below.

The greatest changes after Intralipid were in IL‐6 and CXCL8, both of which play important roles in uterine spiral artery remodelling to facilitate early placental development.^64^ First‐trimester miscarriage is associated with reduced IL‐6 and CXCL8 production,^65^ and lower IL‐6 transcription is evident in the uterine endometrium of women with recurrent miscarriages, compared with fertile women.^66^ Intralipid is potentially responsible for the effect on IL‐6, given that no increase in IL‐6 usually occurs in the luteal phase.^51^, ^67^ Continued elevation in plasma IL‐6 and CXCL8 as pregnancy progresses, indicating excessive inflammatory activation, is linked to preeclampsia^68^ and spontaneous second‐trimester abortion.^69^ The increase in TNF seen in the post‐Intralipid plasma is unlikely to be caused by Intralipid, since TNF is usually elevated to a similar degree in the luteal phase of the menstrual cycle.^51^, ^62^

CCL2 is thought to promote Treg recruitment to the endometrium,^70^ and myeloid‐derived suppressor cells that are increased in early pregnancy are induced by CCL2 in an autocrine manner.^71^ However, a sustained increase in plasma CCL2 is associated with recurrent spontaneous abortion (RSA).^72^ At very high levels, elevated plasma CCL3 is also linked with RSA.^73^ IL‐21 is associated with Th1‐ and Th17‐polarised immune responses, and the 15% increase in median IL‐21 in post‐Intralipid blood samples might reflect elevated production by Th17 cells and NK cells.^74^

Plasma G‐CSF usually declines from the peri‐ovulatory to luteal phase,^60^ increasing the prospect that Intralipid modulates this cytokine. GM‐CSF acts to promote blastocyst survival and implantation competence.^75^, ^76^ Plasma GM‐CSF levels increased in the first trimester of normal healthy pregnancies, but not in women with recurrent miscarriage.^77^ Clinical studies show G‐CSF has therapeutic potential in women with recurrent implantation failure,^78^ potentially via inducing tolerogenic dendritic cells that promote immune tolerance or improve endometrial thickness.^79^

Vascular endothelial growth factor promotes angiogenesis, which is essential for placental development. VEGF is elevated in the endometrial tissue of women who later have successful pregnancy outcome^57^ and is reduced in women with recurrent implantation failure.^80^, ^81^ Serum VEGF does not fluctuate over the cycle but is elevated in the mid‐luteal phase in women undertaking IVF, where it correlates with body mass index and plasma progesterone.^59^ Similar to the current result, a previous study in women showed Intralipid therapy to boost plasma VEGF and CCL2 levels, although this was outside the context of IVF and pregnancy.^82^

Reasonably, it might be expected that if Intralipid exerts beneficial effects on implantation success, higher levels of plasma cytokines and/or greater increases after Intralipid treatment would be seen in the women where embryo implantation and/or progression to live birth occurred. There was no evidence of higher plasma cytokines or a greater change of cytokines in these subgroups, just a more consistent difference between the pre‐Intralipid and post‐Intralipid time points. However, it is important to note that the study was not powered to discriminate effects in these subgroups, so no firm conclusion on associations with reproductive success can be drawn.

The relatively small sample size is a limitation of this study, and we acknowledge that detection of subtle changes would require more study participants. Additionally, the possibility of phenotypic changes in other leucocyte populations was not evaluated here. Although the proportions of white blood cell subsets did not change after Intralipid, effects on function or phenotype of NK cells, macrophages or other innate immune cells that benefit pregnancy are possible. In particular, NK cells are thought to respond to Intralipid therapy and effects on these cells may be important in the setting of recurrent pregnancy loss and repeated implantation failure.^83^

## Conclusion

These data suggest that Intralipid infusion in women receiving treatment for suspected immune‐associated infertility elicits induction of several cytokines linked with systemic immune activation that have potential to improve endometrial receptivity and implantation success. Elevated plasma cytokines were not linked with alterations to circulating T lymphocytes sufficient to implicate the adaptive immune response as being responsive to Intralipid therapy. The data imply that Intralipid treatment may elicit small changes in CD8^+^ Tconv cells but does not affect CD4^+^ T cells including Treg cells. If Intralipid induces immune changes to favor pregnancy success, then the mechanism may involve other immune cell types. The changes outlined in this study warrant confirmation in future studies that include a no‐treatment or sham treatment control.

## Methods

### Ethics approval and study design

Approval for this study was obtained from the human research ethics committee at the University of South Australia, application ID36581, and the scientific advisory committee at Repromed (Dulwich, Australia). Women in good general health undergoing IVF treatment with a frozen–thawed embryo transfer were eligible for inclusion after diagnosis of likely immune‐mediated implantation failure, on the basis of no viable pregnancy despite transfer of multiple good‐quality embryos, together with evidence of serum anti‐nuclear antibodies (ANA) and/or anti‐thyroid antibodies (anti‐thyroglobulin, anti‐thyroid peroxidase) (both from Australian Clinical Labs, Clayton, Australia), and/or elevated endometrial NK cell density.^84^ Exclusion criteria included use of any immune‐modulating drugs (corticosteroids, NSAIDs or methotrexate), positive serology test for hepatitis B, hepatitis C, HIV or syphilis (Australian Clinical Labs), suspected current infection (temperature > 37.5°C on admission) or recent significant physical trauma (injury or surgery). Women were recruited over the 4‐month period from June to September 2017. After obtaining informed written consent, women were administered 100 mL of 20% (v/v) Intralipid (Fresenius Kabi, Sydney, AUS) dissolved in 900 mL of normal saline (Baxter, Sydney, AUS), delivered by intravenous infusion over a 2‐ to 3‐hour period, at approximately 4–5 days post‐ovulation, just prior to embryo transfer. Immediately prior to commencement of Intralipid infusion, 20 mL of peripheral blood (‘pre‐Intralipid’) was collected for cytokine analysis, complete blood leucocyte count and flow cytometric analysis. A second 20‐mL peripheral blood sample (‘post‐Intralipid’) was collected 7–10 days later, in the late‐luteal phase corresponding to the time of embryo implantation. All women then received a second Intralipid infusion, according to the same protocol as the first infusion. Other than Intralipid infusion and peripheral blood collection, the clinical protocol followed standard practice for modified natural cycle frozen embryo transfer,^85^ including administration of Oripro intravaginal micronised progesterone (Orion Laboratories Pty Ltd, Balcatta, WA; 200 mg/day). No other drugs were administered. Clinical outcome was classified initially as ‘embryo implantation’ or ‘no embryo implantation’ on the basis of plasma hCG measured two weeks after embryo transfer. Then, in the event of positive hCG, an ultrasound scan of the uterus was performed in gestational week 7 and week 12. Pregnancy outcome was recorded as early biochemical miscarriage (EBM, before 12 weeks), ectopic pregnancy or live birth (Table 1).

### Plasma collection and isolation of peripheral blood mononuclear cells

Peripheral blood was collected into EDTA‐coated vacutainer tubes, and centrifuged at 2000 × *g* for 10 min at room temperature. Plasma was aliquoted into 500 µL volumes and stored at −80°C until later cytokine analysis by Luminex microbead assay. The remaining blood was diluted in PBS + 2% fetal calf serum (FCS) (Stemcell™ Technologies, Vancouver, CA) (room temperature) to a total volume twice that of the original sample, then overlaid onto Lympholyte (Cedarlane^®^, Ontario, CA) and centrifugation at 600 × *g* for 30 min at room temperature with no brake. The PBMC‐containing buffy coat was collected and washed twice with Dulbecco’s modified PBS (DPBS) by centrifuging at 450 × *g* for 5 min. Cells were resuspended in complete media (RPMI 1640 containing 10% FBS and 1% penicillin/streptomycin solution) and total viable cells counted using a Neubauer haemocytometer. Following counting, cell concentration was adjusted to 4–8 × 10^6^ cells per mL in complete media and diluted in an equal volume of freezing media containing 20% DMSO (Sigma‐Aldrich, NSW, AUS) and 80% FBS (In Vitro Technologies, VIC, AUS), dropwise. Aliquots (1 mL, 2–4 × 10^6^) of PBMCs were then transferred into cryovials and frozen to −80°C overnight, in a Mr Frosty™ (Nalgene^®^, New York, USA) controlled‐rate freezing device. The next day, PBMCs were transferred into vapour phase liquid nitrogen storage until flow cytometry was performed.

### Measurement of cytokines and chemokines in plasma samples

Selected cytokines and chemokines including IL‐2, IL‐4, IL‐10, IL‐12p70, IL‐15 and IL‐21 (Cat. No HTH17MAG‐14K), and CCL2, CCL3, CCL5, CXCL8, CXCL10, G‐CSF, GM‐CSF, GRO, IL‐1RA, IL‐1A, IL‐6, TNF and VEGF (Cat. No HCYTOMAG‐60K) were quantified in plasma samples using Milliplex MAP Luminex microbead assays according to the manufacturer’s instructions (Merck Millipore, Darmstadt, DE). Samples were analysed without dilution in duplicate, and plates were analysed on a Luminex MAGPIX instrument running xPonent v4.2 software (Luminex Corp., Texas, USA). Minimum detectable thresholds were 5.1 pg mL^−1^ (IL‐2), 9.0 pg mL^−1^ (IL‐4), 0.3 pg mL^−1^ (IL‐10), 1.1 pg mL^−1^ (IL‐12p70), 2.7 pg mL^−1^ (IL‐15) and 2.0 pg mL^−1^ (IL‐21), as well as 1.5 pg mL^−1^ (CCL2), 1.9 pg mL^−1^ (CCL3), 1.9 pg mL^−1^ (CCL5), 1.9 pg mL^−1^ (CXCL8), 2.1 pg mL^−1^ (CXCL10), 1.8 pg mL^−1^ (G‐CSF), 3.1 pg mL^−1^ (GM‐CSF), 2.1 pg mL^−1^ (CXCL1), 2.1 pg mL^−1^ (IL‐1RA), 3.3 pg mL^−1^ (IL‐1A), 2.0 pg mL^−1^ (IL‐6), 2.6 pg mL^−1^ (TNF) and 3.7 pg mL^−1^ (VEGF).

### Flow cytometric analysis

Cryopreserved PBMCs were thawed and counted with a haemocytometer; then, 1 × 10^6^ viable cells per sample were rested overnight at 37°C in RPMI 1640 + 10% heat‐inactivated FBS. The following day, cells were stained with viability dye (1/1000 dilution) in PBS with human FCγR block (1/30 dilution) for 10 min. Anti‐CCR7::BUV395 antibody was added and incubated for a further 15 min before adding a master mix of surface‐staining antibodies prepared in Brilliant Staining Buffer Plus (BD Biosciences) (Supplementary table 1). Cells were incubated with antibodies for 20 min, then rinsed and fixed and permeabilised with fix/perm solution (BD Biosciences) for 30 min. After rinsing twice with permwash (BD Biosciences), the cells were incubated with antibodies against intracellular antigens for 40 min, then rinsed with permwash and PBS and finally resuspended in PBS for acquisition with a BD LSR Fortessa X20 flow cytometer. All incubations were performed at room temperature.

### Data and statistical analysis

All data were graphed and analysed with GraphPad Prism for Windows (GraphPad Software, San Diego, California USA, www.graphpad.com (version 9.0.0)). Flow cytometric data were analysed with FlowJo software (BD Biosciences, version 10.6.2). tSNE was performed on a downsample of 3500 pre‐gated CD3^+^ T cells from each sample using default parameters (perplexity = 30, theta = 0.5), according to published protocols.^86^ Xshift clustering was performed with the default Euclidean (*k*) value of 280, as described.^87^ Both tSNE and Xshift were performed in FlowJo software where the proportion of each cluster and MFI of each marker were also calculated. Z‐scores were manually calculated for the MFIs as [(MFI(query) – minimum)/(maximum – minimum)] to generate a heatmap using GraphPad Prism.

## Conflict of interest

The authors have no relevant conflicts to declare.

## Author contribution

**Kerrie Foyle:** Conceptualization; Formal analysis; Investigation; Methodology; Validation; Visualization; Writing‐original draft. **David J. Sharkey:** Conceptualization; Formal analysis; Investigation; Methodology; Project administration; Supervision; Writing‐original draft. **Lachlan Moldenhauer:** Conceptualization; Formal analysis; Investigation; Methodology; Supervision; Writing‐review & editing. **Ella S. Green:** Methodology; Validation; Writing‐review & editing. **Jasmine Wilson:** Formal analysis; Methodology; Validation; Writing‐review & editing. **Cassandra J. Roccisano:** Formal analysis; Investigation; Project administration. **M. Louise Hull:** Conceptualization; Funding acquisition; Investigation; Methodology; Supervision; Writing‐review & editing. **Kelton Tremellen:** Conceptualization; Formal analysis; Funding acquisition; Investigation; Methodology; Project administration; Resources; Supervision; Writing‐review & editing. **Sarah A Robertson:** Conceptualization; Data curation; Formal analysis; Funding acquisition; Investigation; Methodology; Project administration; Supervision; Validation; Writing‐original draft; Writing‐review & editing.

## Supporting information

Supplementary figures 1 and 2
Supplementary tables 1–4
