# Supplementary material for: Effect of Intralipid infusion on peripheral blood T cells and plasma cytokines in women undergoing assisted reproduction treatment
Source: Clin Transl Immunology. 2021 Aug 12;10(8):e1328. doi: 10.1002/cti2.1328 (PMC8358997; doi:10.1002/cti2.1328)
Supplement: Supplementary file 1 — Supplementary figures 1 and 2 Supplementary tables 1–4 [file CTI2-10-e1328-s001.pdf]

Supplementary Information for

Effect of Intralipid infusion on peripheral blood T cells and plasma cytokines in women undergoing assisted reproduction treatment

Running title: Effects of Intralipid on T cells in women

Kerrie L Foyle[1†], David J Sharkey[1†], Lachlan M Moldenhauer[1], Ella S Green[1], Jasmine J Wilson[1], Cassandra J Roccisano[2], M Louise Hull[1], Kelton P Tremellen[3,4], and Sarah A Robertson[1]*.

[1]Robinson Research Institute, Adelaide Medical School, University of Adelaide, Adelaide SA 5005, Australia
[2]School of Pharmacy and Medical Sciences, University of South Australia, Adelaide SA 5001, Australia
[3]School of Medicine, Flinders University, Adelaide SA 5042, Australia
[4]Repromed Pty Ltd, 180 Fullarton Road, Dulwich SA 5065, Australia
[†]Both authors contributed equally to this work

[*]**Corresponding author:** Sarah A. Robertson, The Robinson Research Institute, Adelaide Medical School, University of Adelaide, Adelaide, SA 5005, Australia.
Phone: +61883134094
E-mail: sarah.robertson@adelaide.edu.au

This PDF file includes:

Supplementary figures 1-2
Supplementary tables 1-4

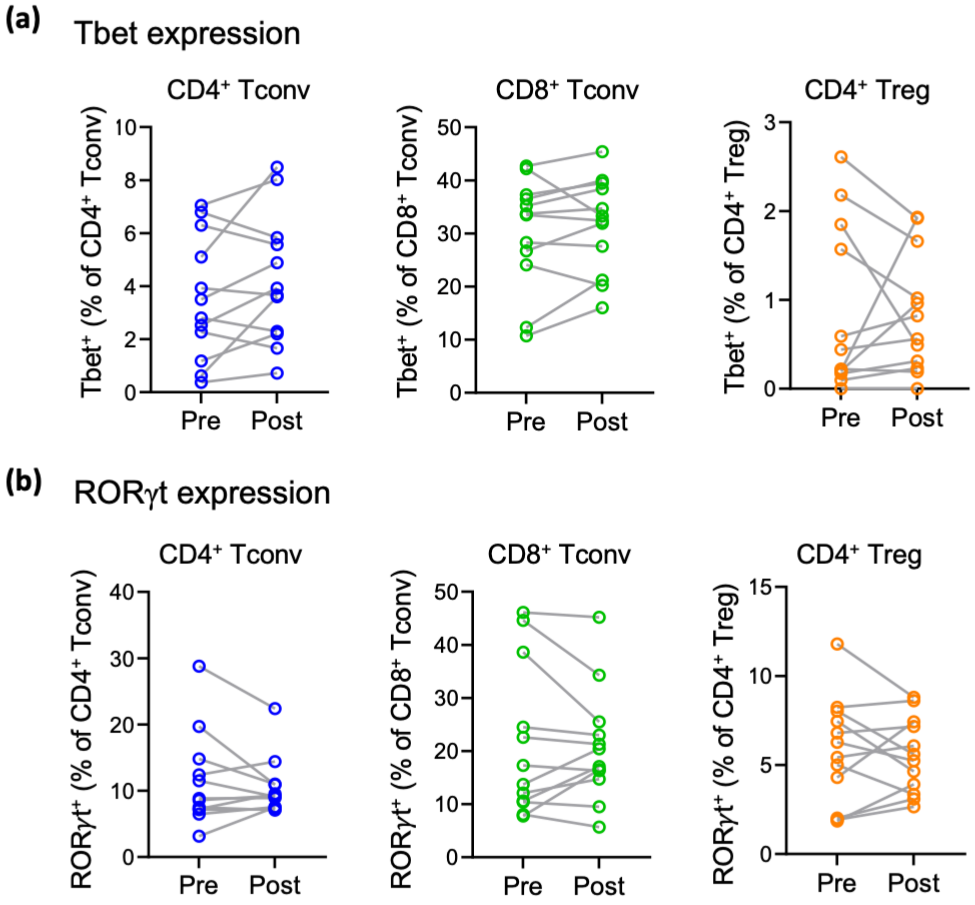

Supplemental Figure S1: Expression of Tbet and RORγt by T cell populations before and after Intralipid treatment. Peripheral blood samples obtained before and after Intralipid treatment were analysed by flow cytometry (N=28 paired samples from N=14 women). Differences in T cell parameters between pre- and post-Intralipid samples were evaluated by the Wilcoxon matched-pairs signed rank test. **(a)** Proportion of Tbet-expressing conventional $CD4^+$ T cells, conventional $CD8^+$ T cells and $CD4^+$ Treg cells. **(b)** Proportion of RORγt-expressing conventional $CD4^+$ T cells, conventional $CD8^+$ T cells and $CD4^+$ Treg cells.

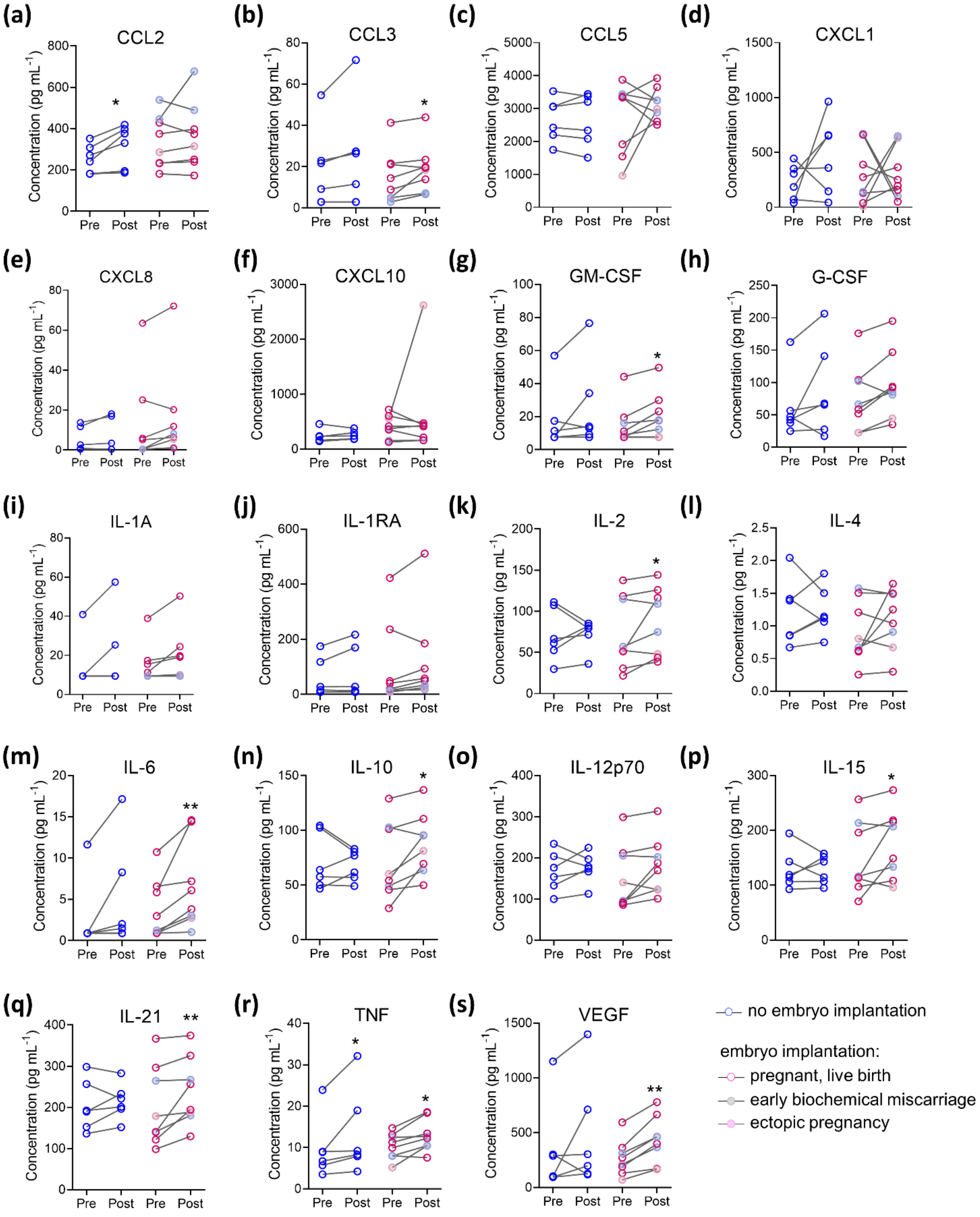

Supplemental figure 2: Effect of Intralipid treatment on plasma cytokine and chemokine levels, according to clinical outcome of ‘embryo implantation’, compared to ‘no embryo implantation’. Plasma from peripheral blood obtained before and after Intralipid treatment was analysed by Milliplex MAP Luminex microbead assay (N=28 paired samples from N=14 women) to quantify CCL2 **(a)**, CCL3 **(b)**, CCL5 **(c)**, CXCL1 **(d)**, CXCL8 **(e)**, CXCL10 **(f)**, GM-CSF **(g)**, G-CSF **(h)**, IL-1A **(i)**, IL-1RA **(j),** IL-2 **(k)**, IL-4 **(l)**, IL-6 **(m)**, IL-10 **(n)**, IL-12p70 **(o)**, IL-15 **(p),** IL-21 **(q),** TNF **(r),** and VEGF **(s)**. Differences in cytokine and chemokine concentrations between pre- and post-Intralipid samples within clinical group were evaluated by the Wilcoxon matched-pairs signed rank test. **P* < 0.05, ***P* < 0.01 and ****P* < 0.005. Cytokine levels prior to and after Intralipid treatment were not different between ‘embryo implantation’, versus ‘no embryo implantation’ groups (Sidak *t*-test). Different symbol colours represent different clinical outcomes: no embryo implantation (blue); embryo implantation resulting in pregnancy and live birth (dark pink); early biochemical miscarriage (grey), or ectopic pregnancy (pale pink).

Supplementary table 1. Flow cytometry antibody details.

Marker	Fluorochrome	Clone	Volume per test (μL)	Supplier
CCR7 (CD197)	BUV395	3D12	2.5	BD Biosciences
CD127	PE-Cy7	HIL-7R-M21	2	BD Biosciences
CD3	APC-H7	SK7	2	BD Biosciences
CD4	BUV496	SK3	2	BD Biosciences
CD8	BUV737	SK1	0.03	BD Biosciences
CD25	BV786	2-A3	2	BD Biosciences
CD45RA	BB515	HI100	2	BD Biosciences
CTLA4 (CD152)	PE-Cy5	BNI3	3.5	BD Biosciences
FOXP3	PE-CF594	236A/E7	2	BD Biosciences
Helios	AlexaFluor 647	22F6	2	BD Biosciences
HLA-DR	BV510	G46-6	2	BD Biosciences
Ki67	APC-R700	SolA15	2	eBiosciences
RORγt	PE	Q21-559	2	BD Biosciences
Tbet	BV421	O4-46	2	BD Biosciences

Supplementary table 2. White blood cell frequencies in women before and after Intralipid therapy.

	pre-Intralipid<br>Median (Range)	post-Intralipid<br>Median (Range)	*P* value[†]
Total white cells	8.04 (4.55 – 19.6)	8.87 (3.58 – 17.4)	0.206
Neutrophils	5.24 (1.78 – 14.86)	6.18 (2.07 – 12.59)	0.148
Lymphocytes	2.27 (1.54 – 3.91)	2.16 (1.01 – 4.09)	0.765
Monocytes	0.39 (0.26 – 0.59)	0.41 (0.16 – 0.56)	0.450
Eosinophils	0.17 (0.02-0.53)	0.13 (0.02 – 0.33)	0.250
Basophils	0.02 (0 – 0.06)	0.03 (0.01 – 0.04)	0.957

[†]Statistical analysis was by the Wilcoxon's matched-pairs signed rank test. N=14 per group.

Supplementary table 3. Frequency of Xshift-defined T cell clusters in peripheral blood of women undergoing IVF before and after Intralipid therapy.

Cluster	pre-Intralipid Median (range)	post-Intralipid Median (range)	*P* value[†]
1	1.5 (0.22 – 5.89)	1.41 (0.16 – 4.81)	0.850
2	8.645 (4.90 – 20.3)	8.82 (5.35 – 22.3)	0.622
3	41.55 (10.8 – 73.1)	38.65 (13.6 – 71.8)	0.746
4	1.655 (0.50 – 6.97)	2.1 (0.30 – 6.83)	0.110
5	1.12 (0.33 – 1.96)	0.98 (0.43 – 2.14)	0.865
6	1.56 (0.25 – 3.65)	1.45 (0.20 – 2.74)	0.424
7	5.02 (1.98 – 13.8)	5.44 (1.51 – 14.7)	0.413
8	1.81 (0.41 – 11.0)	1.40 (0.30 – 9.53)	0.107
9	0.58 (0.03 – 2.12)	0.47 (0.12 – 1.37)	0.458
10	1.02 (0.07 – 15.5)	1.69 (0.18 – 16.1)	0.291
11	0.34 (0 – 21.4)	0.84 (0.03 – 10.8)	0.966
12	3.18 (1.30 – 10.6)	4.08 (2.08 – 12.2)	0.176
13	3.07 (1.03 – 6.54)	2.23 (0.06 – 5.49)	0.519
14	0.49 (0.19 – 2.97)	0.51 (0.10 – 4.11)	0.176
15	1.57 (0.30 – 5.24)	2.13 (0.85 – 6.19)	0.052
16	1.24 (0.37 – 9.90)	1.72 (0.13 – 6.57)	0.392
17	0.21 (0.03 – 1.77)	0.21 (0.03 – 1.73)	0.481
18	1.68 (0.16 – 8.78)	2.05 (0.82 – 8.25)	0.925
19	1.80 (0.18 – 4.30)	2.42 (0.29 – 3.17)	0.850
20	7.46 (2.53 – 14.4)	7.43 (3.99 – 13.0)	0.791

[†]Statistical analysis was by the Wilcoxon’s matched-pairs signed rank test.
N=14 per group.

Supplementary table 4. Concentrations of cytokines and chemokines in plasma samples of women undergoing IVF before and after Intralipid therapy.

Cytokine/ chemokine	pre-Intralipid (pg mL^{-1}) Median (range)	post-Intralipid (pg mL^{-1}) Median (range)	*P* value[†]
CCL2	279 (181 – 539)	352 (173 – 677)	0.042*
CCL3	21.1 (2.9 – 54.7)	19.9 (2.9 – 71.6)	0.001**
CCL5	3057 (959 – 3874)	3095 (1506 – 3922)	0.808
CXCL1	231 (9.9 – 671)	305 (43.8 – 964)	0.391
CXCL8	1.7 (0.4 – 63.5)	5.8 (0.4 – 72.1)	0.012*
CXCL10	295 (130 – 721)	270 (158 – 2620)	0.808
G-CSF	54.3 (22.6 – 176)	83.3 (17.3 – 207)	0.017*
GM-CSF	10.1 (7.5 – 56.9)	15.8 (7.5 – 76.5)	0.010**
IL1A	9.4 (9.4 – 40.9)	9.8 (9.4 – 57.4)	0.016*
IL1RA	23.6 (8.3 – 423)	41.5 (8.3 – 511)	0.027*
IL2	59.3 (21.8 – 138)	79.5 (36.2 – 144)	0.135
IL4	860 (255 – 2041)	1130 (300 – 1801)	0.391
IL6	0.9 (0.9 – 11.6)	3.4 (0.9 – 17.2)	0.001***
IL10	58.7 (28.7 – 129)	78.5 (49.1 – 137)	0.173
IL12p70	147 (85.5 – 299)	176 (101 – 314)	0.104
IL15	116 (70.8 – 257)	146 (95.1 – 274)	0.153
IL21	184 (98.8 – 367)	211 (130 – 374)	0.013*
TNF	9.0 (3.5 – 23.9)	11.3 (4.2 – 32.1)	0.001***
VEGF	242 (69.4 – 1150)	384 (119 – 1398)	0.003**

[†]Statistical analysis was by the Wilcoxon's matched-pairs signed rank test.
N=14 / group. **P* <0.05, ***P* <0.01 and ****P* <0.001.
